# Case Report: Genetic Analysis of a Small Supernumerary Marker Chromosome in a Unique Case of Mosaic Turner Syndrome

**DOI:** 10.3389/fped.2022.799284

**Published:** 2022-02-18

**Authors:** Chao Li, Weiyao Luo, Tingting Xiao, Xingkun Yang, Miaoling Ou, Linghua Zhang, Xiang Huang, Xiaodan Zhu

**Affiliations:** ^1^Department of Prenatal Diagnosis, Foshan Women and Children Hospital Affiliated to Southern Medical University, Foshan, China; ^2^Department of Pediatric, Foshan Women and Children Hospital Affiliated to Southern Medical University, Foshan, China

**Keywords:** Turner syndrome, sSMC, FISH, CNV-seq, del(Y)(q12)

## Abstract

**Background:**

The aim of this study was to explore the source and morphology of a small supernumerary marker chromosome (sSMC) from karyotype analysis of a patient with a unique case of mosaic Turner syndrome. The study findings will provide technical reference and genetic counseling for similar cases.

**Case Presentation:**

A female patient with 46,X,+mar karyotype was diagnosed by genetic karyotype analysis. Genetic methods including fluorescence *in situ* hybridization (FISH) and copy number variation sequencing (CNV-seq) based on low-depth whole-genome sequencing were used to explore the source and morphology of sSMC. FISH technology showed that 56.5% of the cells were X and 43.5% of the cells were XY. CNV-seq detection found that the sSMC was chrY, implying that the patient's karyotype was mos 45,X[58.6%]/46,XY[41.4%]. Retrospective karyotype analysis indicated that the female patient's sSMC was inherited from her father's small chrY. Customized FISH probe of Yq12 microdeletion was positive, indicating that the sSMC was a del(Y)(q12). Based on the results of genetic diagnosis, the specialist doctor gave a comprehensive genetic consultation and ordered regular follow-up examinations.

**Conclusions:**

The findings of the current study showed that the chromosome description of the unique Turner case was mos 45,X[56.5%]/46,X,del(Y)(q12)[43.5%]. FISH technology played a key role in diagnosis of mosaicism. The terminal deletion of mosaic chrY provided a scientific and an accurate explanation for masculinity failure and abnormal sexual development of the current case.

## Introduction

Turner syndrome is a common sex-linked disease. It has the highest incidence of chromosomal abnormalities. The main clinical features of Turner syndrome include short stature, poorly developed secondary sexual characteristics, and infertility. Notably, intelligence of the patient is usually within the normal range. The most common karyotype of this type of patients is 45,X, which represents more than 50% of all cases. The mosaicism karyotype accounts for ~30% of all cases, whereas the mos 45,X/46,XX form accounts for most of the cases. The detection rate of 45,X/46,XY mosaicism, which is a rare chromosomal abnormality and usually not diagnosed in time, is 1.5/10,000 newborns (1). sSMCs refer to a type of extra chromosome fragment outside the normal karyotype. The source of sSMCs cannot be determined by conventional karyotype analysis. In most cases, sSMC's length is less than chr20, and may be derived from any chromosome. sSMCs are present in ~3.3 million human beings currently (2). The other population (~1.2 million) of sSMC carriers are clinically affected either by adverse effects of gained genetic material present on the sSMC or by uniparental disomy of the sSMC's sister chromosomes (2). Patients with sSMCs have variable clinical phenotypes owing to differences in the genetic material present in the marker chromosomes (3). These sSMCs cause uncertainty in clinical symptoms and make genetic diagnosis and counseling challenging. Therefore, it is important to accurately locate the source and morphology of sSMC. Currently, genetic diagnosis techniques are diverse. High-throughput sequencing methods have become more popular, such as CNV-seq and whole exome sequencing (WES). These methods are more comprehensive and accurate compared with traditional karyotyping and FISH probes. However, traditional technologies play irreplaceable roles. In the current study, an sSMC carried by a special mosaicism Turner syndrome was determined through a variety of mainstream molecular cytogenetic methods. Moreover, the source and morphology of the sSMC was successfully identified. The findings obtained through genetic analysis and interpretation of the current case provide scientific and technical reference for similar cases. Furthermore, the findings supplement effective genetic evidence and provide useful insights for abnormal sexual development.

## Materials and Methods

### Study Subject

An 8-year-old patient showed stunted growth for more than 3 years. The patients visited the hospital for clinical consultation. The patient had no history of chronic diseases and had normal mental capacity. The height of the father of the patient was 170 cm and the height of the mother was 159 cm. The patient was born by cesarean section by a G2P2 mother. The patient did not have a history of asphyxia. The birth weight was 3.0 kg and the height was 52 cm. It showed that the patient was in good spirits and had normal reactions by physical examination. The patient has a childish face and a sharp voice, showed tanner stage I of vulva and genitalia, B1 breast score, and was in PH1 stage. Pelvic examination through B-ultrasound showed a uniform uterine muscular echo; only the right breast was developed, and no obvious ovarian echo was detected in the appendages on both sides. Pituitary MRI plain scan and enhanced scan did not show any abnormalities. GnRHa provocation test (including levodopa and arginine compound) was performed after the outpatient clinic. The initial diagnosis of endocrinology specialist was growth hormone deficiency and Turner syndrome. The female patient was then referred to the center of prenatal diagnosis for disease diagnosis and genetic counseling.

### Methods

#### Karyotype Analysis of Peripheral Blood

Karyotyping is able to detect polyploidy, aneuploidy, translocations, inversions, rings, and copy number changes in the size range of 4–6 Mb. Lymphocyte culture medium was purchased from Da Hui Biology Company, Beijing. The patient signed an informed consent form, and peripheral blood was aseptically drawn and inoculated into the culture medium. Cell culture, harvest, G banding, and slide production were carried out in turn. G-banding slides were scanned and analyzed using the GSL120 platform, Leica Biosystems. The 5 karyotypes were analyzed under the microscope and 20 cells were counted. The count was expanded to 50 metaphase cells if mosaicism existed. Reporting of karyotype results was described based on the nomenclature system of human cytogenetics (ISCN-2016) (4).

#### Fluorescence *in situ* Hybridization

There are FISH probes for chromosomes 13, 18, 21, X, and Y, which may be used for rapid detection of aneuploidy prior to formal karyotyping. In addition, there are multiple FISH probes for areas associated with microdeletion. FISH probe was purchased from Jin Pu Jia Medical Technology Company, Beijing (5). Peripheral blood was drawn after the patient signed the informed consent. The experiment was performed following the manufacturer's instructions. After preparation of cell suspension, slide pretreatment, denaturation, and hybridization, slides were washed and sealed in turn. Samples were then analyzed by microscopy. Each set of probes randomly counted 50 cells. The count was expanded to 100 or 200 cells if more than one cell with abnormal signal was detected. If the proportion of abnormal cells in a certain indicator was ≥10%, it indicated that the indicator was abnormal. If the proportion of abnormal cells was between 10 and 60%, it indicated the presence of mosaicism (6).

#### Low-Depth Whole-Genome Sequencing

CNV analysis has become a first-tier clinical cytogenetics procedure in patients with unexplained developmental delay/intellectual disability. Peripheral blood was drawn after the patient signed the informed consent. DNA was extracted with an automatic nucleic acid extraction system. Library was prepared using the Ke Nuo An kit purchased from Bei Rui company, Beijing. Original read files were obtained by sequencing on the HiSeq3000 platform. Sequencing files were compared with the human reference genome GRCH37/hg19 using the bowtie2 package in Linux (7). Samtools package in Linux was used to sort and make index to obtain the bam intermediate file (8). The wisecondorx package in Linux was then used to calculate and visualize CNVs of each chromosome band of the samples (9). Genetic interpretation of the results on CNVs was performed following the 2020 version of ACMG and ClinGen's latest interpretation guidelines (10).

#### Screening for AZF Microdeletion of ChrY

PCR multiplex assays are the method of choice for quickly revealing genomic microdeletions in the large repetitive genomic sequence blocks on the long arm of the human chrY. They harbor the Azoospermia Factor (AZF) genes, which cause male infertility when functionally disrupted. The ChrY microdeletion kit was purchased from Tou Jing Biotechnology Company, Beijing. The kit was used to qualitatively detect whether the AZF that affected sperm production on the chrY had a microdeletion. This fragment was located in the Yq band. Multiplex PCR-capillary electrophoresis technology was used for detection. A total of 15 sequence-tagged sites (STS) were selected following international guidelines. Primers were designed, and 4 tubes were used for multiplex PCR detection. In addition, the sex-determining region of chrY (SRY) (Yp11.3) was detected. All experiments and result interpretation were performed following the instructions in the kit.

## Results

### Chromosome Karyotype Analysis

**Figure 1:**
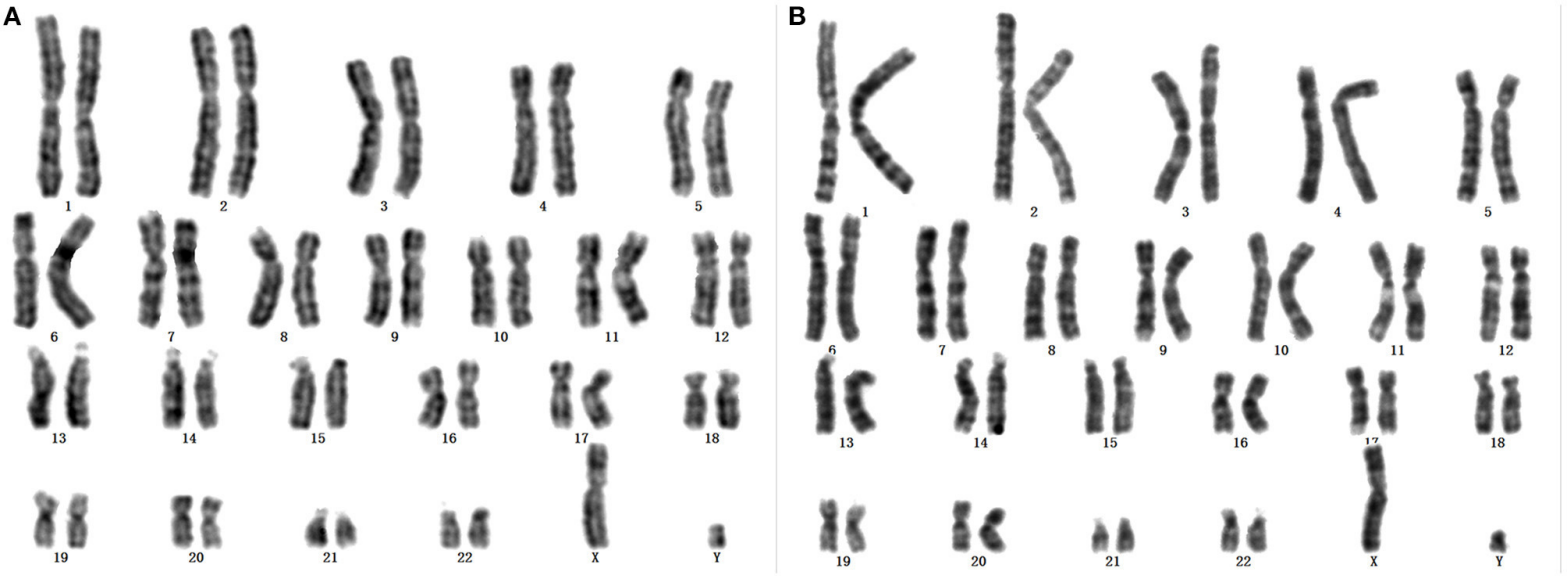
**(A)** Karyotype of the proband: 46, X, +mar. **(B)** Karyotype of father to the proband: 46, X, Yq-.

Karyotypes of the proband and her father were 46, X, +mar and 46, X, Yq-, respectively (Figures 1A,B). Analysis of family members showed that the morphology of +mar was probably inherited from the father's chrY. The chrY of the father occurred as a very small fragment, which may be due to normal shortening of the heterochromatin region of Yq12 or loss of pathogenicity of the other band of chrY. The social sex of the proband was female, and further analysis was needed to clarify the source and morphology of the +mar.

### Fluorescence *in situ* Hybridization

**Figure 2:**
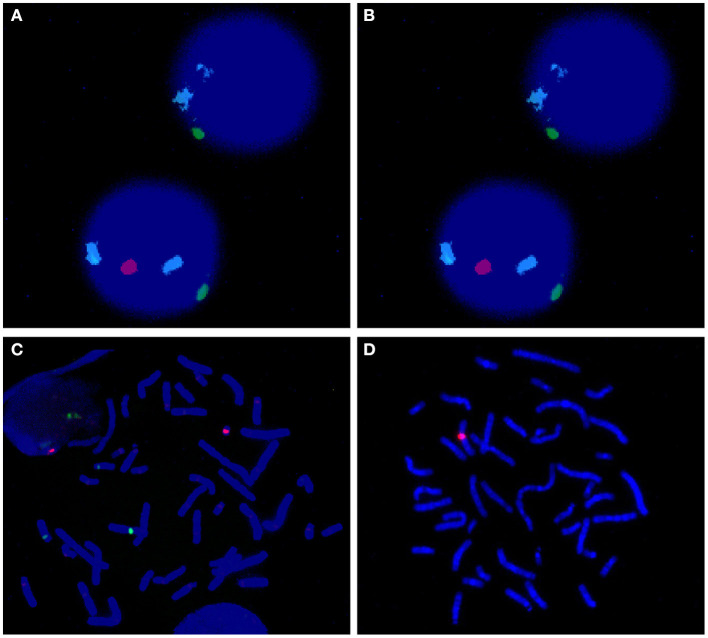
**(A)** FISH of uncultured interphase cell; azure: chr18; green: chrX; red: chrY. The upper cell image shows no chrY whereas the lower cell image shows presence of chrY. **(B)** FISH of cultured metaphase cell; azure: chr18; green: chrX; red: chrY. These findings showed that no chrY was ppresent (implying that only X was present). **(C)** FISH of cultured metaphase cell; azure: chr18; green: chrX; red: chrY. These findings showed presence of chrY (implying presence of XY). **(D)** Yq12 FISH of cultured metaphase cell; red: chrX; green:chrY. The findings showed Yq12 deletion.

Chromosome centromeric probes of FISH CSPX/CSPY (green/red) using uncultured peripheral blood sample showed that 56.5% of the cells had a single green signal (indicating X). Furthermore, 43.5% of the cells showed one green and one red signal (indicating XY) when 200 cells were counted (Figure 2A). Analysis of the cultured peripheral blood samples showed that 18.0% of the 200 cells had a green signal (indicating X) (Figure 2B). Notably, 82.0% of the 200 cells showed a green signal and a red signal (indicating XY) (Figure 2C). The FISH probe of Yq12 microdeletion showed that chrX only had a red signal, which was used as a normal positive control. In addition, all metaphase cells did not show a green signal, indicating that Yq12 was completely deleted (Figure 2D).

### Detection of Microdeletion and Microduplication

**Figure 3:**
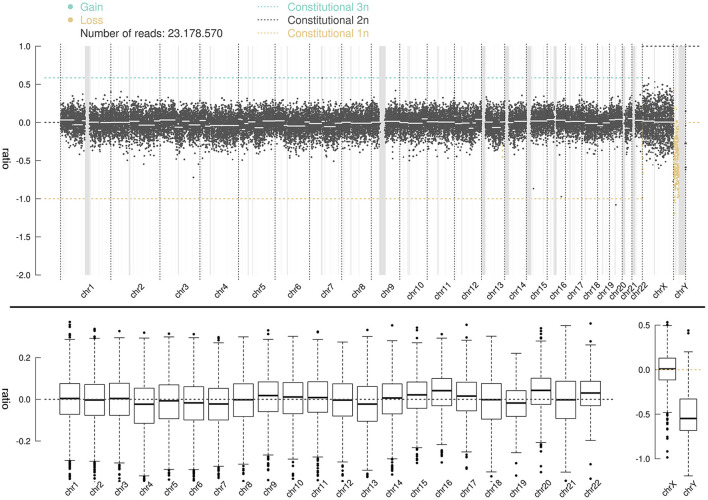
CNV-seq distribution map for 46 chromosomes; the ordinate ratio represents lo${\text{g}}_{2}\frac{test}{2}$; the test is the copy number of the corresponding band. The abscissa represents the chromosome band. A ratio of −1 indicates that one copy is lost; a ratio of 0 represents a normal value; a ratio of 0.58 indicates that one copy is gained; a ratio between −1 and 0 represents a mosaic deletion, and the ratio of abnormal cells is ~2 (1-2^ratio^).

Whole-genome high-throughput sequencing results showed that the sSMC was chrY (Figure 3), and the interval ratio of Yp11.3-Yq11.223 was ~-0.5. Calculation and conversion indicated that the patient's karyotype was a mosaic deletion of 45,X[58.6%]/46,XY[41.4%]. Screening of the AZF of chrY did not show abnormality of microdeletion in the AZF region (Yq) and SRY gene region (Yp11.3).

### Outcome and Follow-Up

Because Turner syndrome can involve multiple organ systems, the risk of occurrence of some complications increases with age. The patients face different neuropsychological problems at different age groups. According to the genetic detection results, the laboratory actively communicated with a clinical specialist. The specialist notified the family members to return to the outpatient clinic to further improve the relevant system examinations, and used estrogen and progesterone drugs to treat symptomatically in a timely manner in order to improve the patient's final height and maintain secondary sexual characteristics, so that the uterus can develop normally; various complications also can be prevented. Ask the patient for regular specialist follow-ups to monitor diseases of related systems or organs such as gonadotropin, cardiovascular, urinary system, liver and kidney function, eyes and ears, and autoimmunity. It was recommended that the patient maintained a healthy and active lifestyle and took medications actively. The specialist also informed her medical progress about the disease, including the possibility of prenatal diagnosis or assisted reproduction in later stages.

## Discussion and Conclusion

The World Health Organization reports that the incidence of birth defects is ~6.42% in low-income countries, 5.57% in middle-income countries, and 4.72% in high-income countries. Rapid development of molecular genetic technologies such as next-generation sequencing enables detection of more genomic abnormalities. Prevention and control of birth defects and genetic counseling have been widely advocated in the society.

In the current study, the morphology and source of sSMC carried by a rare mosaicism Turner syndrome were identified through a comprehensive application of various mainstream molecular cytogenetics techniques. Karyotyping technique was used to explore the sSMC of unknown origin. Family traceability was conducted to determine whether the sSMC's morphology was similar to the small chrY of the proband's father. FISH technology and advanced high-throughput sequencing technology were then used to confirm that the sSMC of the female proband was actually the chrY. High-throughput sequencing results showed that chrY was reduced. However, due to the limitations of the methods, it was not possible to determine the specific chromosome description. Therefore, it was not clear whether the reduction was due to mosaicism being X/XY or it was due to the Y band loss (11).

Moreover, the abnormality of the centromere and heterochromatin region of each chromosome was not detected owing to the presence of the detection blind band. Therefore, high-throughput sequencing could not detect the abnormality of the Yq12 region. Conventional normal FISH probe, which is a standard method for judging the specific proportion of mosaicism cells, confirmed the karyotype to be mosaicism X/XY. The findings of normal FISH demonstrated that chrY reduction was caused by mosaicism detected through high-throughput sequencing. Although sSMC was identified as the source of the chrY, its length was too short, and further analysis was performed to determine which bands were lost. Therefore, customized Yq12 probe of FISH was used to explore whether there was a deletion at the end of chrY. The findings showed that the mosaicism X/XY was a del(Y)(q12). We searched related literatures and found some research reports: 42.86% (6/14) of the mosaic patients were mosaic for a structurally abnormal chrY in 46,XY cell lines; three with Yqh-, one with a Yq deletion, one with a Yp+, and the last with a dicentric Y. Four of these patients also presented with AZF microdeletions (12). The clinical phenotype of 45,X/46,XY individuals is very broad and includes Turner females, varying degrees of genital malformations, and men with normal phenotypes (13). The gonads of such patients have been reported as streak gonads, ovarian-like, and/or exhibiting other histopathological abnormalities in previous case reports, small studies, and large multicenter histological studies (14, 15).

These findings show that the nature of sex determination may be determined by expression and regulation of related genes of chrY. Defective chrY results in the person presenting a female phenotype even though that person possesses a karyotype of XY. This results in defective testicular development; thus, the infant may or may not have fully formed male genitalia internally or externally. Full range of ambiguity of structure may occur, mainly if mosaicism is present. The child is usually a girl with the features of Turner syndrome or mixed gonadal dysgenesis if the Y fragment is minimal and non-functional (16, 17). This is consistent with findings of the current study; thus, it explains the current case. However, the presence of small fragment variation and point mutations about sex determination-related genes in the sSMC in the case cannot be ruled out owing to the limitations of CNV-seq. Therefore, further studies should be performed. A genetic counselor carrying out genetic counseling of similar patients could include WES analysis based on the patient's wishes and economic conditions.

In summary, a comprehensive tracing analysis of an sSMC carried by a unique mosaicism Turner syndrome was explored through a variety of mainstream molecular cytogenetic methods, and the source and morphology of the sSMC was successfully identified. The findings of the current unique case provide scientific and accurate technical reference for similar cases. Furthermore, these findings supplement effective genetic evidence and provide useful insights into abnormal sexual development.

## Data Availability Statement

The original contributions presented in the study are included in the article/supplementary material, further inquiries can be directed to the corresponding author/s.

## Ethics Statement

The study was approved by the Science and Technology Bureau of Foshan City, Guangdong Province, China. All subjects had signed an informed consent statement, agreeing that the data may be used for non-profit scientific research and teaching purposes. This study complies with the hospital's ethical review regulations.

## Author Contributions

The research idea was derived from XZ and XH. CL designed the experiments. XY participated in collecting the data. WL and LZ analyzed the data. CL and TX wrote the paper. All authors had read and approved the final manuscript.

## Funding

This study was funded by the Engineering Technology Center of Precision Diagnosis of the Genetic Disease of Foshan City, Project Number: 2020001003953. The role of the funding was to support the cost of the experiment and language editing of the paper.

## Conflict of Interest

The authors declare that the research was conducted in the absence of any commercial or financial relationships that could be construed as a potential conflict of interest.

## Publisher's Note

All claims expressed in this article are solely those of the authors and do not necessarily represent those of their affiliated organizations, or those of the publisher, the editors and the reviewers. Any product that may be evaluated in this article, or claim that may be made by its manufacturer, is not guaranteed or endorsed by the publisher.
